# Gamma probe and ultrasound-guided percutaneous localisation of the sentinel lymph node in breast cancer patients

**DOI:** 10.1186/bcr3255

**Published:** 2012-11-09

**Authors:** P Whelehan, A Evans, S Vinnicombe, D Brown, D McLean

**Affiliations:** 1 University of Dundee, UK

## Introduction

A major reason for failure to diagnose axillary lymph node metastasis preoperatively in breast cancer patients is that needle biopsy may not target the sentinel node (SLN). We aimed to address this by testing the accuracy with which we could identify and target the sentinel lymph node percutaneously under combined radioisotope and ultrasound guidance.

## Methods

Ethical approval was obtained. In 48 patients scheduled for surgical sentinel lymph node biopsy (SLNB), following injection of radioisotope, one of three radiologists used a gamma probe in tandem with ultrasound guidance to identify a SLN prior to surgery and mark it with a localising wire. The patients then proceeded to surgical SLNB, guided by radioisotope and blue dye. The surgeon noted whether the wire had correctly marked a SLN.

## Results

The SLN was correctly localised in 75% of patients (36 of 48; 95% CI = 63 to 87%). A learning curve for the three operators was observed, with a rate of correct localisation in the first five procedures performed by each operator of 67% (10 of 15) rising to 79% in the subsequent 33 (26 of 33; 95% CI = 64 to 93%). Interoperator variations in accuracy were evident, with a success rate of 83% in the most experienced of the three.

## Conclusion

Percutaneous SLN localisation using combined radioisotope and ultrasound guidance is feasible. Use of this method to guide needle biopsy of the axilla could increase the preoperative diagnosis rate for axillary lymph node metastases in breast cancer patients.

